# Early-Stage Induction of SWI/SNF Mutations during Esophageal Squamous Cell Carcinogenesis

**DOI:** 10.1371/journal.pone.0147372

**Published:** 2016-01-26

**Authors:** Hidetsugu Nakazato, Hideyuki Takeshima, Takayoshi Kishino, Emi Kubo, Naoko Hattori, Takeshi Nakajima, Satoshi Yamashita, Hiroyasu Igaki, Yuji Tachimori, Yukio Kuniyoshi, Toshikazu Ushijima

**Affiliations:** 1 Division of Epigenomics, National Cancer Center Research Institute, Tokyo, Japan; 2 Esophageal Surgery Division, National Cancer Center Hospital, Tokyo, Japan; 3 Department of Thoracic and Cardiovascular Surgery, Graduate School of Medicine, University of the Ryukyus, Okinawa, Japan; 4 Endoscopy Division, National Cancer Center Hospital, Tokyo, Japan; Baylor University Medical Center, UNITED STATES

## Abstract

The SWI/SNF chromatin remodeling complex is frequently inactivated by somatic mutations of its various components in various types of cancers, and also by aberrant DNA methylation. However, its somatic mutations and aberrant methylation in esophageal squamous cell carcinomas (ESCCs) have not been fully analyzed. In this study, we aimed to clarify in ESCC, what components of the SWI/SNF complex have somatic mutations and aberrant methylation, and when somatic mutations of the SWI/SNF complex occur. Deep sequencing of components of the SWI/SNF complex using a bench-top next generation sequencer revealed that eight of 92 ESCCs (8.7%) had 11 somatic mutations of 7 genes, *ARID1A*, *ARID2*, *ATRX*, *PBRM1*, *SMARCA4*, *SMARCAL1*, and *SMARCC1*. The *SMARCA4* mutations were located in the Forkhead (85Ser>Leu) and SNF2 family N-terminal (882Glu>Lys) domains. The *PBRM1* mutations were located in a bromodomain (80Asn>Ser) and an HMG-box domain (1,377Glu>Lys). For most mutations, their mutant allele frequency was 31–77% (mean 61%) of the fraction of cancer cells in the same samples, indicating that most of the cancer cells in individual ESCC samples had the SWI/SNF mutations on one allele, when present. In addition, a BeadChip array analysis revealed that a component of the SWI/SNF complex, *ACTL6B*, had aberrant methylation at its promoter CpG island in 18 of 52 ESCCs (34.6%). These results showed that genetic and epigenetic alterations of the SWI/SNF complex are present in ESCCs, and suggested that genetic alterations are induced at an early stage of esophageal squamous cell carcinogenesis.

## Introduction

Genetic alterations, such as somatic mutations, are deeply involved in human carcinogenesis by disrupting various cancer-related pathways [1–6]. Recent whole-exome sequencing has highlighted the role of disruption (inactivation) of the SWI/SNF chromatin remodeling complex, which regulates gene transcription by mobilizing nucleosomes [7, 8]. Various components of the SWI/SNF complex are frequently mutated in various types of cancers. *ARID1A* is frequently mutated in ovarian clear cell carcinomas [9, 10], hepatocellular carcinomas (HCCs) [11, 12], and gastric cancers [4, 6, 13]; *ARID2* in HCCs [11, 12, 14]; *PBRM1* in renal cell carcinomas [15]; and *SMARCA4* in small cell carcinomas of the ovary of hypercalcemic type (SCCOHT) [16–18]. As for esophageal squamous cell carcinomas (ESCCs), somatic mutations have been detected for *ARID1A*, *ARID2*, and *PBRM1* by exome-sequencing [3].

The components of the SWI/SNF complex are also inactivated by aberrant DNA methylation of promoter CpG islands [13, 19], which is known to be involved in the repression of gene transcription. Components of the SWI/SNF complex, *ACTL6B*, *SMARCA2*, and *SMARCD3*, and those of the other types of chromatin remodeling complex, *ATRX* and *SMARCA1*, are aberrantly methylated in gastric cancers [13]; *ARID1A* in invasive breast cancers [19]; *ARID1B* in pancreatic cancers [20], and *ACTL6B* in hepatocellular carcinomas (HCCs) [21]. However, the presence of aberrant methylation of the components of the SWI/SNF complex in ESCCs is still unclear.

In this study, we aimed to clarify, in ESCC, 1) what components of the SWI/SNF complex have somatic mutations by deep sequencing using a bench-top next generation sequencer to overcome the intrinsic limitation in the reading depth of exome-sequencing, 2) what components have aberrant methylation, and 3) when somatic mutations of the SWI/SNF complex occur. It was found that genetic and epigenetic alterations of the SWI/SNF complex are present in ESCCs, and it was suggested that genetic alterations are induced at an early stage of esophageal squamous cell carcinogenesis.

## Materials and Methods

### 2.1 Clinical samples

Ninety-two primary ESCC samples and their corresponding non-cancerous tissue samples were endoscopically collected from ESCC patients with written informed consents. The collected samples were stored in RNAlater (Life Technologies, Carlsbad, CA, USA) at -80°C until the extraction of genomic DNA. Clinical information of the 92 ESCCs is listed in Table 1. The study was approved by the Institutional Review Boards of the National Cancer Center. Genomic DNA was extracted from ESCC samples by the standard phenol/chloroform method, and was quantified using a Quant-iT PicoGreen dsDNA Assay Kit (Life Technologies).

**Table 1:** Clinicopathological data of the ESCC samples. Clinical T, N, and M stages were based upon the 7th edition tumor-node-metastasis (TNM) classification of the International Union Against Cancer (UICC). SCC, squamous cell carcinoma.

Characteristics	Categories	No. of patients
Total		92
Age		30–79 (average, 64.3)
Sex	Male	79
	Female	13
Tumor site	Upper	11
	Middle	55
	Lower	26
Histology	SCC	92
Clinical T stage	T1a	2
	T1b	22
	T2	11
	T3	56
	T4	1
Clinical N stage	N0	22
	N1	42
	N2	23
	N3	5
Clinical M stage	M0	71
	M1	21

### 2.2 Cell lines

Nine human ESCC cell lines, KYSE30, KYSE140, KYSE170, KYSE180, KYSE220, KYSE270, KYSE410, KYSE450, and KYSE510, were obtained from the Japanese Collection of Research Bioresources (JCRB) Cell Bank [22]. Two neuroblastoma cell lines, IMR-32 and KELLY, were obtained from the JCRB Cell Bank and Public Health England, respectively. KYSE140 was cultured in Ham's F12 medium containing 2% (v/v) FBS; KYSE30, KYSE170, KYSE180, KYSE220, KYSE270, KYSE410, KYSE450, and KYSE510 were cultured in Ham's F12/RPMI1640 medium containing 2% (v/v) FBS; IMR-32 was cultured in MEM medium containing 10% (v/v) FBS and non-essential amino acid (NEAA); and KELLY was cultured in RPMI1640 medium containing 10% (v/v) FBS.

### 2.3 Analysis of somatic mutations

Mutation analysis of 18 genes encoding components of the SWI/SNF complex was conducted as described previously [13]. Briefly, a DNA library containing 672 kinds of DNA fragments covering 86.5–100% (mean 96.9%) of the coding regions of the 18 genes (*ACTL6A*, *ACTL6B*, *ARID1A*, *ARID1B*, *ARID2*, *ATRX*, *PBRM1*, *PHF10*, *SMARCA1*, *SMARCA2*, *SMARCA4*, *SMARCAL1*, *SMARCB1*, *SMARCC1*, *SMARCC2*, *SMARCD1*, *SMARCD3*, and *SMARCE1*) was prepared by multiplex PCR. A DNA library prepared from an ESCC sample was uniquely barcoded, and sequencing was conducted using an Ion Proton Sequencer (Life Technologies).

The sequences obtained were mapped onto the human reference genome (hg19). Somatic mutations in individual ESCC samples were identified by subtraction of the sequence variations also detected in the corresponding non-cancerous tissue of the cancer sample. Somatic mutations identified using the Ion Proton Sequencer were confirmed by Sanger sequencing of amplified DNA using the primers listed in S1 Table. As for mutations with a low frequency, amplified DNA was cloned into pGEM-T Easy vector (Promega, Madison, WI, USA), and sequences were confirmed by analysis of 10 pools of four clones (40 clones).

### 2.4 Analysis of DNA methylation

DNA methylation data of primary ESCCs and ESCC cell lines were obtained using an Infinium HumanMethylation450 BeadChip array (Illumina, San Diego, CA), which covered 482,421 CpG sites in a previous study (GSE74693) [23]. Among various CpG sites, only those in TSS200 [a region between transcription start site (TSS) and its 200 bp upstream] or 1st exon/5'-UTR with CpG islands were analyzed for *ACTL6A*, *ACTL6B*, *ARID1A*, *ARID2*, *PBRM1*, *SMARCA2*, *SMARCA4*, *SMARCAL1*, *SMARCB1*, *SMARCC1*, *SMARCC2*, *SMARCD1*, *SMARCD3*, and *SMARCE1* as described previously [13]. DNA methylation was assessed using β values, and genes were defined as unmethylated (β value, 0–0.2), partially methylated (β value, 0.2–0.4 for primary ESCCs and 0.2–0.8 for ESCC cell lines), and methylated (β value, 0.4–1.0 for primary ESCCs and 0.8–1.0 for ESCC cell lines).

DNA methylation levels of *ACTL6B* in non-cancerous esophageal tissues were analyzed by quantitative methylation-specific PCR (qMSP) as described previously [24], using primers listed in S2 Table.

### 2.5 Analysis of a cancer cell fraction in an ESCC sample

The cancer cell fraction of an ESCC sample with mutation(s) of the SWI/SNF complex was analyzed by measuring DNA methylation levels of three genomic regions, *TFAP2B*, *ARHGEF4*, and *RAPGEFL1*, which are specifically methylated in ESCC cells [23]. The highest methylation level of the three genomic regions was defined as the cancer cell fraction, as described previously [23]. The eight ESCC samples had cancer cell fractions of 23–87% (mean 54%) (S3 Table).

### 2.6 Expression analysis

Genome-wide gene expression analysis was conducted using a GeneChip Human Genome U133 Plus 2.0 expression microarray (Affymetrix, Santa Clara, CA), as described previously [25, 26]. Obtained signal intensity of an individual probe was normalized so that mean signal intensity of all the probes would be 500. Mean signal intensity of all the probes in an individual gene was defined as its transcription level, and genes with 250 or more of signal intensities were defined as expressed genes [25].

Gene-specific expression of *ACTL6B* in ESCC cell lines and non-cancerous esophageal tissues was analyzed by quantitative RT-PCR as described previously [25], using primers listed in S2 Table. IMR-32 and KELLY were used as positive controls with *ACTL6B* expression based upon the findings in the Cancer Cell Line Encyclopedia (CCLE) [27].

### 2.7 Statistical analysis

The association between SWI/SNF alterations, namely SWI/SNF mutations and *ACTL6B* methylation, and tumor characteristics, namely clinical T stage, clinical N stage, and clinical M stage, was evaluated by the Fisher exact test.

## Results

### 3.1 Various components of the SWI/SNF complex were mutated in ESCCs

**Fig 1:**
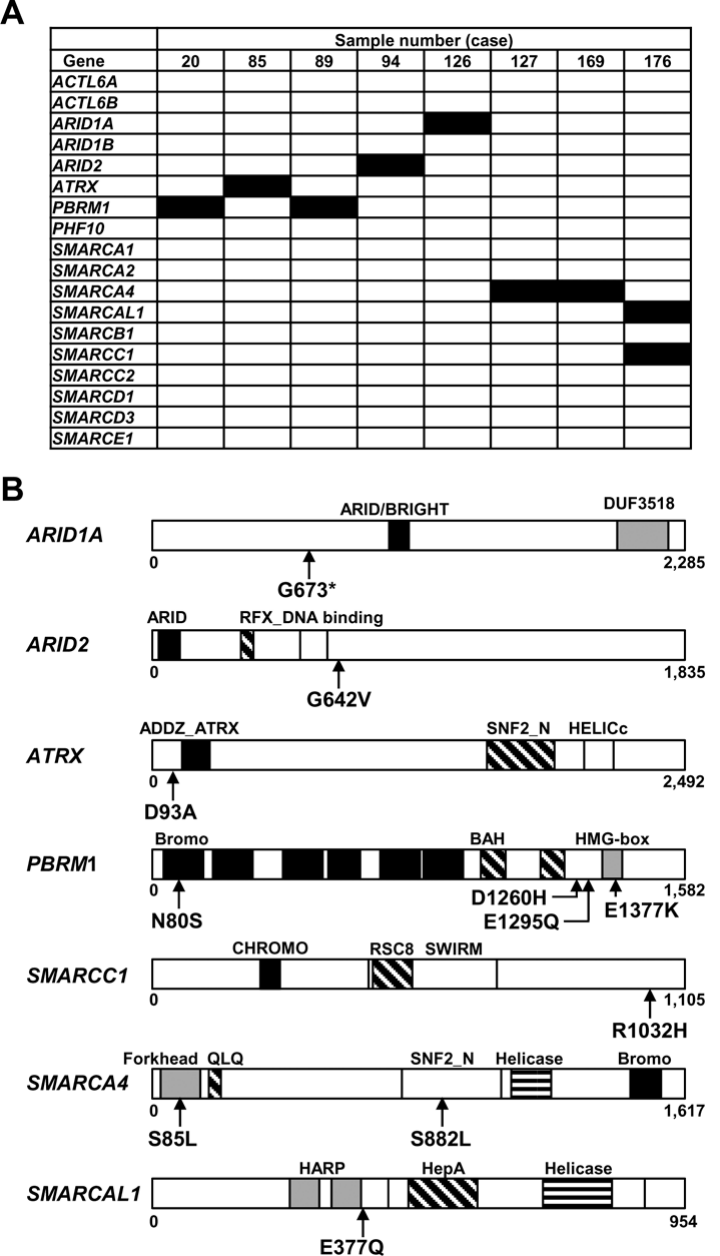
Somatic mutations of genes encoding the components of the SWI/SNF complex in ESCCs. (A) Status of somatic mutations of the SWI/SNF complex in ESCCs. Somatic mutations were analyzed in the 92 ESCCs by an Ion Proton Sequencer. Among the 92 ESCCs, 8 (8.7%) had 11 somatic mutations of 7 genes, *ARID1A, ARID2, ATRX, PBRM1, SMARCA4, SMARCAL1* and *SMARCC1*. Filled box indicates the presence of somatic mutations. The presence of these somatic mutations was confirmed by Sanger sequencing. (B) The position of somatic mutations in the components of the SWI/SNF complex. Somatic mutations were located in various functional domains of a mutated component.

Ninety-two ESCC samples were analyzed by amplicon sequencing using a bench-top next generation sequencer for 18 genes encoding components of the SWI/SNF complex (mean reading depth = 1,369). Eight of the 92 ESCCs (8.7%) had 11 somatic mutations of 7 genes, *ARID1A*, *ARID2*, *ATRX*, *PBRM1*, *SMARCA4*, *SMARCAL1*, and *SMARCC1* (Table 2, Fig 1A and 1B). *SMARCA4* (2 mutations in 2 ESCCs) and *PBRM1* (4 mutations in 2 ESCCs) were mutated in multiple ESCCs, and other genes were mutated in one ESCC. Among these mutations, mutations of *ATRX* (16.3%) and *ARID2* (10.6%) showed low allele frequencies but were able to be successfully detected by deep sequencing (1,191 reads for *ATRX* and 765 reads for *ARID2*). Six of the nine ESCC cell lines had potential somatic mutations for *ARID1A*, *ARID2*, *ATRX*, *PHF10*, *SMARCA1*, and *SMARCA4* (S4 Table).

**Table 2:** Somatic mutations detected in the 92 ESCCs. Termination codon is shown by *. Novel mutations in ESCCs are marked by #.

Case	Gene	Read coverage	Mutant allele frequency (%)	Nucleotide change	Amino acid change
20	*PBRM1*	439	70.8	c.239A>G	Asn80Ser
85	*ATRX*	1191	16.3	c.277G>A	Asp93Asn
89	*PBRM1*	403	64.3	c.4129G>A	Glu1377Lys
	*PBRM1*	1427	67.4	c.3883G>C	Glu1295Gln
	*PBRM1*	567	64.4	c.3778G>C	Asp1260His
94	*ARID2*	765	10.6	c.1925G>T	Gly642Val
126	*ARID1A*	669	80	c.2017C>T	Gln673*
127	*SMARCA4*	504	26	c.2644G>A	Glu882Lys
169	*SMARCA4*	838	29	c.254C>T	Ser85Leu
176	*SMARCAL1*^*#*^	3384	58	c.1129G>C	Glu377Gln
	*SMARCC1*^*#*^	3795	47.8	c.3095G>A	Arg1032His

The somatic mutations were located in various functional domains (Fig 1B). The *SMARCA4* mutations were located in the Forkhead (85Ser>Leu) and SNF2 family N-terminal (882Glu>Lys) domains. The *PBRM1* mutations were located in the bromodomain (80Asn>Ser) and the HMG-box domain (1377Glu>Lys). The presence of these somatic mutations was confirmed by Sanger sequencing. These results showed that various genes encoding components of the SWI/SNF complex were mutated in ESCCs.

### 3.2 Somatic mutations were present in most cancer cells in individual ESCCs

**Fig 2:**
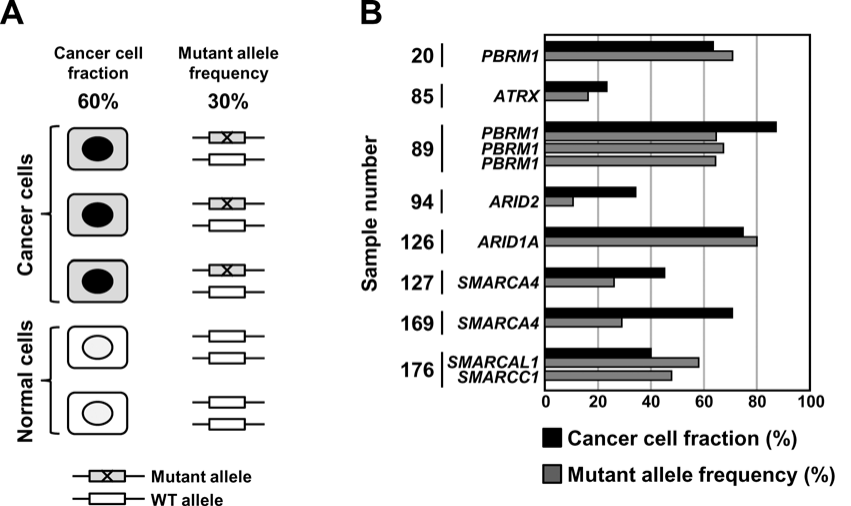
The close association between a cancer cell fraction and the mutant allele frequency in the ESCC samples. (A) The association of a cancer cell fraction and a mutant allele frequency in a cancer sample. Theoretically, in the case that all the cancer cells in an individual cancer sample have a somatic mutation on one allele of a specific gene and allelic imbalance of the region is absent, a mutant allele frequency is expected to be 50% of the cancer cell fraction. (B) The cancer cell fraction of ESCCs. The cancer cell fraction was analyzed using cancer cell fraction markers, *TFAP2B, ARHGEF4,* and *RAPGEFL1,* which are specifically methylated in ESCC cells. The mutant allele frequency was calculated using read numbers of sequences with and without somatic mutations. The mutant allele frequency of the five ESCC samples (#85, #89, #94, #127, and #169) was 31–77% (mean 61%) of their cancer cell fraction.

To analyze the timing of the somatic mutations of the SWI/SNF complex, a cancer cell fraction was estimated for each of the eight ESCC samples with mutation(s) of the SWI/SNF complex, and the association between the fraction and mutant allele frequency was analyzed. Theoretically, in the case that all the cancer cells in an ESCC sample have a somatic mutation on one allele of a specific gene and allelic imbalance of the region is absent, a mutant allele frequency is expected to be 50% of a cancer cell fraction (Fig 2A). The mutant allele frequency of five of the eight ESCC samples (#85, #89, #94, #127, and #169) was lower than their cancer cell fraction in the same samples, and ranged from 31 to 77% (mean 61%) of the cancer cell fraction (Fig 2B). In contrast, the mutant allele frequency of the other three ESCC samples (#20, #126, and #176) was higher than their cancer cell fraction, and ranged from 107% to 145% (mean 121%) of the cancer cell fraction (Fig 2B). This result showed that most of the cancer cells in some ESCC samples had SWI/SNF mutations on one allele, and suggested that somatic mutations of the SWI/SNF complex are induced at an early stage of esophageal cell carcinogenesis.

### 3.3 Aberrant DNA methylation of *ACTL6B* was present in ESCCs

**Fig 3:**
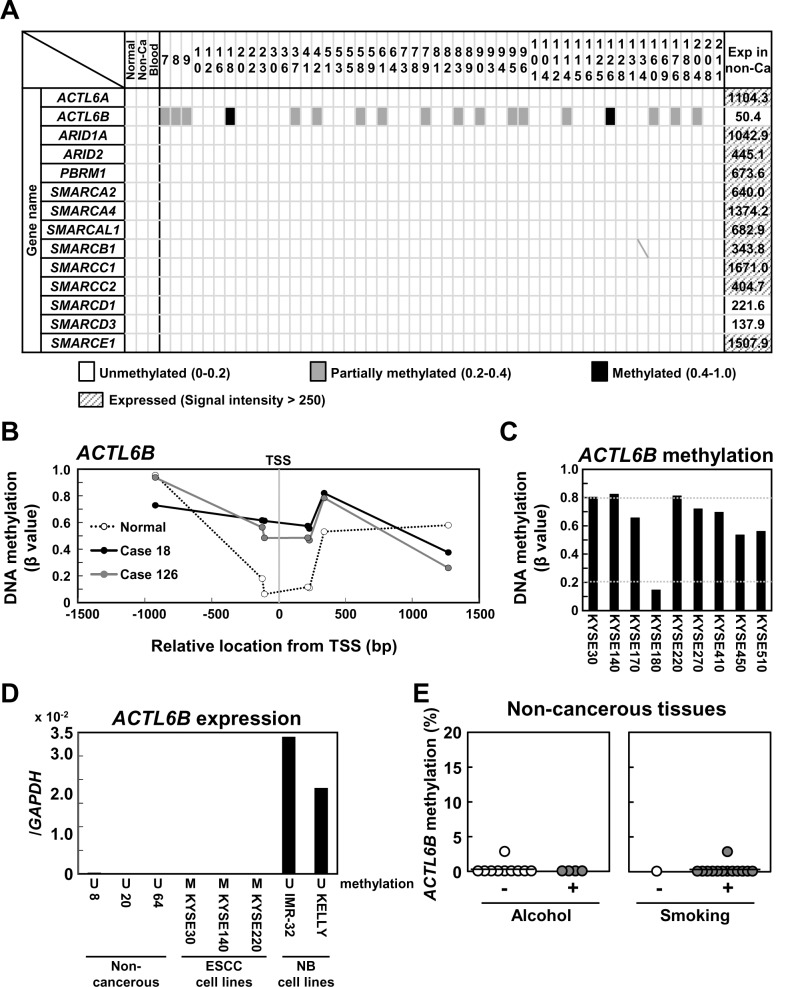
Aberrant DNA methylation of the components of the SWI/SNF complex in ESCCs. (A) Status of DNA methylation of the SWI/SNF complex in ESCCs. DNA methylation was analyzed in the 52 ESCCs by an Infinium HumanMethylation450 BeadChip array. Among the 52 ESCCs, 18 (34.6%) had aberrant methylation of *ACTL6B*. The expression level of each gene in non-cancerous esophagus tissues (n = 8, pooled) is shown in the rightmost of the panel. (B) DNA methylation status of *ACTL6B* around TSS. Aberrant methylation was induced around TSS of *ACTL6B*. (C) DNA methylation of *ACTL6B* in ESCC cell lines. Three ESCC cell lines, KYSE30, KYSE140, and KYSE220, had complete methylation of *ACTL6B*. (D) Expression levels of *ACTL6B* in ESCC cell lines and non-cancerous esophageal tissues. *ACTL6B* was not expressed in ESCC cell lines with its complete methylation (KYSE30, KYSE140, and KYSE220), and was expressed in neuroblastoma cell lines (NB) without its methylation (IMR-32 and KELLY). This supported that *ACTL6B* methylation could be involved in its silencing in tissues where it is expressed. At the same time, *ACTL6B* was not expressed in non-cancerous esophageal tissues without its methylation. This suggested that *ACTL6B* methylation was a passenger in esophageal squamous cell carcinogenesis. DNA methylation status of non-cancerous tissues and cell lines was analyzed by qMSP and an Infinium HumanMethylation450 BeadChip array, respectively. (E) DNA methylation of *ACTL6B* in non-cancerous esophageal tissues. *ACTL6B* was not aberrantly methylated in non-cancerous tissues regardless of alcohol/smoking exposure.

DNA methylation data were available from our previous study for 52 of 92 ESCCs [23]. Eighteen of the 52 ESCCs (34.6%) had aberrant methylation of *ACTL6B* at its promoter CpG island, but normal esophageal sample and non-cancerous tissue sample did not (Fig 3A and 3B). As for the other components of the SWI/SNF complex, *ACTL6A*, *ARID1A*, *ARID2*, *PBRM1*, *SMARCA2*, *SMARCA4*, *SMARCAL1*, *SMARCB1*, *SMARCC1*, *SMARCC2*, *SMARCD1*, *SMARCD3*, and *SMARCE1*, none of the 52 ESCCs had their aberrant methylation (Fig 3A). Three ESCC cell lines, KYSE30, KYSE140, and KYSE220, had a completely methylated *ACTL6B* promoter (Fig 3C), and *ACTL6B* was not expressed in these cell lines (Fig 3D). In contrast, two neuroblastoma cell lines, IMR-32 and KELLY, had an unmethylated *ACTL6B* promoter, and *ACTL6B* was expressed. These results supported that *ACTL6B* methylation could be involved in its silencing in tissues where it is expressed.

To assess the role of aberrant DNA methylation of *ACTL6B* in esophageal squamous cell carcinogenesis, *ACTL6B* methylation and expression were analyzed in non-cancerous esophageal tissues. *ACTL6B* was unmethylated, but was not expressed (Fig 3A and 3D). This result suggested that *ACTL6B* methylation was a passenger in esophageal squamous cell carcinogenesis.

The association between alcohol/smoking exposure and aberrant DNA methylation of *ACTL6B* was analyzed in non-cancerous esophageal tissues. *ACTL6B* was not aberrantly methylated in non-cancerous tissues, regardless of alcohol/smoking exposure (Fig 3E).

### 3.4 SWI/SNF alterations were not associated with characteristics of ESCCs

The association between somatic mutations of the SWI/SNF complex, also *ACTL6B* methylation, and tumor characteristics was analyzed. Neither somatic mutations of the SWI/SNF complex nor aberrant *ACTL6B* methylation was associated with clinical T stage, clinical N stage, and clinical M stage (Table 3). This result showed that SWI/SNF alterations were not associated with characteristics of ESCCs.

**Table 3:** The association between SWI/SNF alterations and clinicopathological characteristics.

		SWI/SNF mutation			*ACTL6B* methylation		
Characteristics	Categories	(+)	(-)	*P* value	(+)	(-)	*P* value
Total		8	84		18	34	
Clinical T stage				0.71			1
	T1 and T2	2	33		6	12	
	T3 and T4	6	51		12	22	
Clinical N stage				1			0.41
	N0	2	20		3	3	
	N1, N2, and N3	6	64		15	31	
Clinical M stage				0.38			0.73
	M0	5	66		13	27	
	M1	3	18		5	7	

## Discussion

Components of the SWI/SNF complex, *ARID1A*, *ARID2*, *ATRX*, *PBRM1*, *SMARCA4*, *SMARCAL1*, and *SMARCC1*, were mutated in ESCCs. Among these, somatic mutations of *SMARCAL1*, and *SMARCC1* were identified for the first time in ESCCs. Somatic mutations with low allele frequencies were successfully detected by deep sequencing. This suggested that deep sequencing focusing on specific sets of genes is useful to detect somatic mutations with low allele frequencies, which are generally difficult to detect by whole-exome sequencing.

Early-stage induction of alterations of the SWI/SNF complex during carcinogenesis has also been suggested for cancers other than ESCCs. During esophageal adenocarcinoma (EAC) development, somatic mutations of *ARID1A* and *SMARCA4* are already present in benign metaplastic never-dysplastic Barrett's esophagus (NDBE) [28]. During gastric carcinogenesis, aberrant methylation of an ISWI component, *SMARCA1*, was detected in normal gastric tissues of people infected with *Helicobacter pylori* [13], a potent gastric cancer inducer. These early induction of genetic and epigenetic alterations of chromatin remodeling factors in multiple types of cancers suggested that their inactivation may be involved in predisposition to cancers (the formation of a field for cancerization [29]).

The mutant allele frequencies of three ESCCs (#20, #126, and #176) were higher than their cancer cell fractions. Theoretically, in the case that all the cancer cells in an ESCC sample have a somatic mutation on one allele of a specific gene and allelic imbalance of the region is absent, a mutant allele frequency is expected to be 50% of a cancer cell fraction (Fig 2A). Therefore, these three ESCCs might have a copy number loss of the wild type allele and this might result in the higher mutant allele frequency than cancer cell fractions.

Aberrant DNA methylation of promoter CpG islands is generally known to cause silencing of their downstream genes [30]. Regarding *ACTL6B*, aberrant methylation was found in its promoter CpG island, and the island was methylated in ESCCs. Expression analysis in cell lines supported that *ACTL6B* could be silenced by aberrant methylation of its promoter CpG island. At the same time, *ACTL6B* was not expressed in non-cancerous esophageal tissues, which had an unmethylated *ACTL6B* promoter. Therefore, *ACTL6B* methylation was considered to be a passenger in esophageal squamous cell carcinogenesis. In contrast, somatic mutations of other components of the SWI/SNF complex were likely to be drivers because the genes with the mutations were expressed in non-cancerous esophageal tissues (Fig 3A).

Mechanistically, disruption of the SWI/SNF complex has been reported to repress cell growth in other types of cancers [6, 13]. Therefore, it is likely that inactivation of the SWI/SNF complex is involved in esophageal squamous cell carcinogenesis by promoting cell growth rate. At the same time, the SWI/SNF complex is known physiologically to regulate a large number of genes that are involved in a wide variety of cancer-related pathways, including the Wnt pathway, the p53 pathway, the MAPK pathway, DNA repair, cell cycle regulation, and apoptosis [31]. Therefore, complicated combinations of disruption of multiple cancer-related pathways might be alternative mechanisms of esophageal squamous cell carcinogenesis.

In conclusion, genetic and epigenetic alterations of the SWI/SNF complex are present in ESCCs, and genetic alterations were suggested to have been induced at an early stage of esophageal squamous cell carcinogenesis.

## Supporting Information

S1 Table Primers used for Sanger sequencing. (XLSX)

S2 Table Primers used for DNA methylation analysis and expression analysis. (XLSX)

S3 Table Cancer cell fractions in the 8 ESCC samples with mutations of chromatin remodelers. (XLSX)

S4 Table Potential somatic mutations detected in the 9 ESCC cell lines. (XLSX)
